# When It Comes to Screen Golf and Baseball, What Do Participants Think?

**DOI:** 10.3390/ijerph192013671

**Published:** 2022-10-21

**Authors:** Bo-Hyun Seong, Chang-Yu Hong

**Affiliations:** 1 Chungbuk Research Institute, Cheongju-si 28517, Chungcheongbuk-do, Korea; 2 Division of Global and Interdisciplinary Studies, Pukyong National University, Busan 48513, Korea

**Keywords:** virtual reality sports game, perceived usefulness, perceived ease of use, intention to use, technology acceptance model

## Abstract

Screen golf and baseball activities have been popular as virtual game content and sport activities, but no one has cogently explained why they are attractive to Korean urban society. Our research team analyzed the decision-making process for participating in screen golf and baseball through a widely used technology acceptance model (TAM) to explain the relationship between perceived ease of use, perceived usefulness, personal attitude, and individual intention. Structural equation modeling (SEM) verified five hypotheses established through a literature review, and 400 effective samples obtained through online surveys provided material for analysis. As a result of the analysis, perceived usefulness was the most important variable leading to participation in virtual reality sports. Based on this finding, we could conclude that the successful popularization of virtual reality sports depends on the development of applications sophisticated enough to provide practical usefulness to participants, such as physical posture correction and an improvement in personal athletic skills.

## 1. Introduction

Due to the impacts of COVID-19, the reality expansion market, which includes AR and VR, has been rapidly growing since 2021, and related hardware sales are expected to grow to USD 270 billion in 2025 (www.StrategyAnalytics.com (9 July 2020)). Gartner, a research company, announced in its 2017 Hype Cycle Report that the applications of virtual reality technology are rapidly expanding. In addition, Virtual Reality (VR) has become attractive to multiple industries as a useful instrument for shopping, education, sports training, entertainment and leisure [1].

Digitization has significantly changed the number of people who join physical athletic and sports activities [2]. In particular, as the technology develops and hardware devices become cheaper, VR is being used as a solution to bestow an optimal sports experience [1]. These days, myriad sports games have been developed that provide users with various benefits, including fitness care, game playing, and physical training [3,4,5,6,7], and this trend is advancing at a rapid pace [8].

South Korea is making outstanding achievements in the VR sports industry due to a rapidly advancing and high-quality IT infrastructure. According to the Korea Intellectual Property Office [9], patent technology developed to improve virtual reality sports is significantly increasing every year. The future growth potential for convergence technologies, such as virtual reality sports games, can be inferred from patent applications alongside other documented information in the field [10]. According to the patent application rate for each virtual reality sports event registered by 2018, 33% of applications were for golf (107 cases) and 16% for baseball (67 cases). The high percentage of patent applications for screen golf and screen baseball emphasizes their significance as subjects of this study.

Screen golf and screen baseball are the result of high-tech IT technologies such as precision sensors that are capable of detecting fine movements of players, golf clubs, baseball bats and balls, and high-definition projectors that can show virtual reality reproductions of various outdoor golf courses and baseball fields [8,11,12]. In particular, Korea’s leading IT enterprise, which highly values its sustainability in the future, recognizes the golf industry as an opportunity for growth and is participating in the industrialization competition [8]. By the end of 2020, South Korea’s screen golf market exceeded USD 1.1 billion, laying the foundation for the industrialization of screen golf.

On the other hand, the screen baseball market experienced a severe recession during the COVID-19 pandemic. This change is in evident contrast to the situation before 2020, when screen baseball was expected to lead the growth of the virtual reality sports market in Korea. Although various simple statistical analyses have been made of this, no academic approach has been made for generalized discussions based on relevant theory. In particular, although screen baseball seems to be similar to screen golf—both are sports games combined with virtual reality technology—we posit that the factors affecting the decision-making process of continuing to use screen golf and screen baseball may be different. Simply put, baseball is a team sport and golf is an individual sport activity.

Perhaps because few countries have attained advancement and improvement in industrializing virtual reality sports games, there has not been much academic investigation into the decision-making process leading to participation. The rapid advancement of Korean sports VR is a rare achievement, but that rarity inhibits study, such that limited research has been carried out on screen golf or baseball [8,12,13,14].

In the era of the Fourth Industrial Revolution, products and content combined with ideas from various fields of IT are constantly being produced, and many scholars are calling for more research on AR and VR [15,16,17]. Due to this, consumer acceptance should be discussed at present, when convergence with newly developed information technology is occurring in various fields. Although technology acceptance models (TAM) are widely used to explain the decision-making process of consumers who accept new technologies [18,19,20], the application of such research to virtual reality sports games is difficult to find.

TAM is regarded as the most effective, yet concise and straightforward, model for describing consumers’ cutting-edge technology adoption procedures [8]. In related research, increased investment in information technology in the 1990s, as well as rapid expansion in personal computer distribution and Internet use, contributed to the widespread adoption of technology acceptance models [21,22,23,24,25,26]. The value of the technology acceptance model became more generally acknowledged in the 2000s as the usage of high-tech products and services in everyday life increased. The cause of the success and failure of the two events may be determined from the standpoint of consumer acceptance by essentially addressing the difference in the decision-making process of screen golf and screen baseball by the user through the primary factors of the technology acceptance model.

Based on this, this study attempts to focus on the following research issue: what factors contribute to the success or failure of virtual reality sports such as screen golf and screen baseball? The purpose of this study is to apply the technology acceptance model as a theoretical framework for solving research issues, and was carried out to examine variations in the decision-making process of users by two groups, using the influence relationship between main variables. Although the screen golf market continues to grow despite the COVID-19 pandemic, screen baseball is reported to be in recession. This divergence suggests that there may be differences in the decision-making process for the use of each sport.

Based on a relevant literature analysis, such as an analysis of the literature concerning virtual reality sports games and TAM, the necessity of this study was discussed and hypotheses were established. Through a survey conducted with adult participants possessing a wide range of experience using screen golf and screen baseball, hypothesis verification and results were tested and analyzed, and theoretical and practical implications and future research tasks were extrapolated.

## 2. Conceptual Note and Literature Review

### 2.1. Virtual Reality Sports

Technological progress and the rapid expansion of the IT industry are driving changes in production and consumption patterns in various fields of modern society [27]. In sport, digital technologies are mainly applied to measure, analyze or broadcast the performances of real-world athletes [28]. However, in recent years, a number of sports applications have emerged that pursue a more holistic approach, encompassing health and play, by offering their users a wide range of features [4,6,7,29].

The application of virtual reality technology in sports is significant because it expands opportunities to meet potential demand by overcoming temporal and environmental constraints [8]. Screen golf and screen baseball, which are popular in Korea, are representative examples. VR is a 3D environment created by computers, which allows for users to explore and interact, and VR worlds are implemented such that users have a similar experience to reality through one or more of the five senses [30,31,32,33,34]. VR sports are sports implemented by applying VR technology to produce an experience as if the user is participating in actual sports activities. Although it may vary between sports and sporting events, VR sports collectively refers to a system combining hardware (e.g., motion platform, beam projector) that detects the movement of equipment and participants with software to implement the movement of equipment and participants on the screen.

Unlike general VR games, VR sport games implement real sports, so the verisimilitude of users’ experience is as a primary factor. In previous studies, ‘reality’ was explained as a psychological state in which users exist and are active in a VR environment [35,36,37,38,39], suggesting that the ultimate goal is to reach a state in which they do not feel as if they are participating in virtual reality.

South Korea is one of the societies leading the growth of the global virtual reality sports market thanks to high-speed internet environment and excellent advanced information technology (IT) [8]. In Korea, the Intellectual Property Office (IPO) reported that various derivative VR sectors are being formed by applying sensing and display technology from screen golf—which leads the virtual reality sports market with approximately USD 1 billion in sales in 2017—to baseball, running, cycling, shooting, soccer, bowling, tennis, fishing, skiing, and curling [9]. 

**Figure 1:**
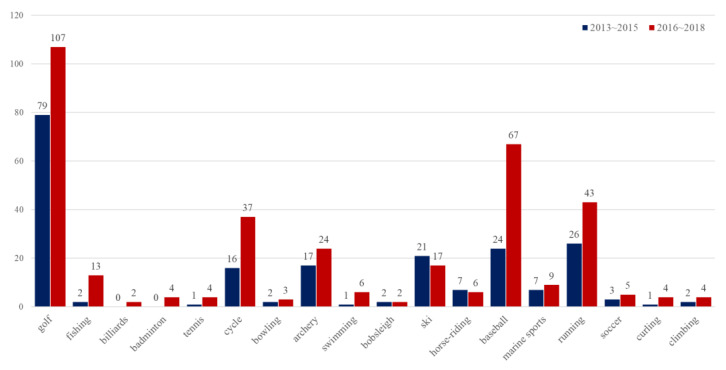
Current status of patent applications by virtual reality sports event. Source: Korean Intellectual Property Office’s Press release (22 May 2019).

According to the Korean IPO (Figure 1), there have been 357 patent applications in the field of virtual reality sports over the past three years (2016–2018). This is a 69% increase from the 211 applications (2013–2015) over the previous three years, and the growth of screen golf and screen baseball-related technologies is particularly noteworthy [9]. The fact that the number of patent applications for screen golf and screen baseball is much higher than that of other sports reflects the popularity of these two events.

The development of the two virtual reality sports technologies shows that competition between related companies is intensifying as purveyors seek to increase the market share. Actually, screen golf and screen baseball franchises are located all over South Korea, in both residential and commercial areas, and they are usually composed of individual rooms for from three to four people, creating communal venues for leisure time with friends and colleagues. However, the fact that negative impacts of the COVID-19 pandemic are largely confined to the screen baseball market prompts research into consumer behaviour.

People who intimately enjoy outdoor golf and baseball in neighborhood public parks, such as North American societies, might describe the differences between screen golf and baseball in a casual or dismissive manner, but these authors undertook this research work to scrutinize their subtle psychological distinctions. Screen golf and screen baseball have several things in common. First, both are operated as franchises in most cities across the country, allowing for ease of access. Second, since both are indoor sports activities, they can be played without constraints from seasonal weather conditions. Third, since it is possible to play alone or with a small number of people, the stress on a requisite number of people is low. Fourth, the cost is relatively cheaper than real golf and baseball. 

**Figure 2:**
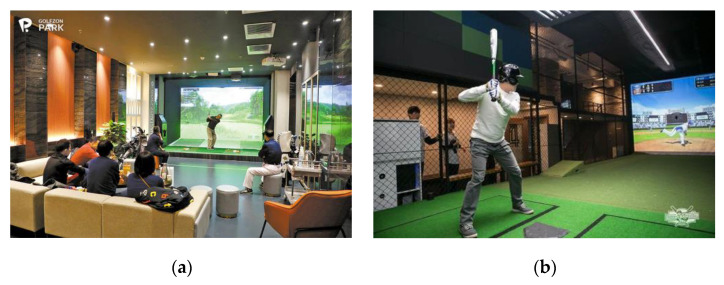
Screen golf (**a**) and baseball (**b**) facilities. Source: https://www.joongang.co.kr/article/24018948#home (24 March 2021) (**a**), https://www.segye.com/newsView/20180701002828 (2 July 2018) (**b**).

In screen golf, users can play the entirety of 18 actual courses, but screen baseball differs in that users can only play the simple act of hitting a ball flying from a batting machine. In other words, screen golf produces an experience closer to real golf. Conversely, screen baseball differs significantly in that it can only exercise batting for the offensive team—the computer program is responsible for the offensive team’s base running and all of the defensive team’s actions (See Figure 2).

The following are the technical specs for screen golf and screen baseball. Sensors that can properly monitor the movement of the ball, which is an important part of the game, have been created in both events, and mistakes have been eliminated by carefully measuring the user’s posture using motion plates. It also boasts high-quality visual and sound systems, such as those seen on genuine golf courses and baseball stadiums. In order to continue with the game, a touch monitor kiosk is built.

### 2.2. Technology Acceptance Model (TAM)

Preceding the advent of VR sports, attempts have been and continue to be made to apply new information technologies in nearly every field of work and entertainment. Therefore, various theories are used in the field of social psychology to describe the decision-making process through which consumers choose products with new technologies, including the theory of reasoned action [40], theory of planned behavior [41], innovative diffusion model [42], and TAM [43]. Among them, the TAM is accepted as the most explanative yet concise model for elucidating the process of consumers’ accepting new technologies [8]. As a result, TAM has been applied in various contexts and has received empirical support from numerous studies [44,45].

TAM is a model for quantitatively predicting and explaining factors that influence people’s acceptance of information technology; the model is based on Fishbein and Ajzen [40]’s theory of reasoned action and explains the effects of two variables: perceived ease of use and perceived usefulness of information technology on users’ intention or attitude [43]. In the field of information technology, TAM is recognized as a model with high explanatory power for investigating the process of consumer acceptance of new technologies [26,46,47,48,49].

The technology acceptance model is presented as major variables, such as perceived ease of use, perceived usefulness, attitude, and intention, and is a concise theory that explains the decision-making process by which new products and services created with cutting-edge information technology are accepted by consumers. ‘Perceived ease of use’ refers to the degree to which users expect certain information technologies or systems to provide a straightforward operation without much mental and physical effort [43]. Davis [43] defined perceived usefulness as “the subjective probability that future users believe will improve their job performance in the organization by using a specific application system,”, which is based on Bandura’s [30] theory of self-efficacy. This is because the easier it is for people to adopt a new technology or service, the more likely they are to see it as useful.

In other words, if the technique of usage is so simple that an individual does not feel uneasy about embracing new technology, this has a good influence on the usability of the technology, developing a favorable attitude. A pleasant attitude would influence the intention to use positively, suggesting a sequence of processes that lead to real action (the use of items and services) [43,50]. Several subsequent studies have reevaluated the concentrated validity and discriminant validity of these concepts and confirmed the relationship between them [51,52,53].

In the 1990s, the use of TAMs mainly focused on the personal use of new information systems in corporate working environments [54,55,56,57,58]. On the other hand, in the 2000s, the focus was on identifying variables affecting the use of goods and services featuring IT enhancement in daily life and leisure activities [18,20,47,59,60,61,62,63,64,65,66,67].

Recently, many researchers have become accustomed to discussing the user’s acceptance process for e-learning, a parallel example in which information technology is applied in the educational field [68,69,70,71,72]. Due to the COVID-19 pandemic, TAM-related research is expected to be conducted more widely in the educational field. Since infectious diseases are anticipated to become commonplace, increased application of information technology and public use of its essential elements are crucial for society. For similar reasons, the popular use of information technology in the leisure sports field is accelerating, but, unlike in the education field, there are relatively few related discussions on the use of TAM concerning VR sports.

## 3. Research Methodology

### 3.1. Research Model and Hypotheses

We designed a conceptual model to test five related hypotheses in this study. Hypotheses one through four show the relationships among perceived ease of use, perceived usefulness, attitude, and intention. To address the lack of academic research on the formation of participation decision and purchase behaviors related to screen golf and screen baseball, which exhibits the combination of advanced IT with general activity, this study adopted TAM due to its superior ability to explain decision-making processes/behaviors.

The direct influence relationship between perceived ease of use, perceived usefulness of use, and attitude, which are key variables constituting the technology acceptance model, has already been verified through previous studies [44,47,73,74,75,76,77]. Therefore, this study established the following hypotheses to investigate the structural causal relationship between perceived ease of use, perceived usefulness, and attitude based on previous studies:

**Hypothesis** **1.** *Perceived ease of use has a significant positive effect on perceived usefulness*.

**Hypothesis** **2.** *Perceived ease of use has a significant positive effect on attitudes*.

**Hypothesis** **3.** *Perceived usefulness of use has a significant positive effect on attitudes*.

Behavioral intentions refer to a person’s willingness to conduct a certain action [39]. As suggested in various theories of consumer behavior, such as TAM, TPB, and TRA, intention is a variable that can predict behavior more accurately than attitude [40,41,43,78]. Therefore, intention is the most accurate variable that can predict behavior [40,79], and it is common either to set it as the last dependent variable in the research model instead of behavior or to set it in a mediating role between attitude and behavior [80]. Based on this, hypothesis four was established:

**Hypothesis** **4.** *Attitude has a significant positive effect on behavioral intention*.

In addition, hypothesis five was established to explore whether there is a difference in the influencing relationship between variables in the process leading to participation in screen golf and screen baseball:

**Hypothesis** **5.** *There is a difference in the influence relationship between variables in the process leading to participation in screen golf and screen baseball*.

### 3.2. Data Collection and Analytic Design

The population of the study was narrowed to consumers over the age of 20 in Korea who had experience playing screen golf or screen baseball. Convenience sampling was used as the sample method; survey respondents between their 20s and 40s, which are important as potential demand for expanding the market, were quite over-represented in the sample compared to the general population, as this was the dominant age group of participants (see Table 1). The online survey was conducted from September 2021 to January 2022, using computer-assisted web interviewing supported by a private survey company with master samples of 428,000 people with a similar proportion to the domestic census population.

As established above, online surveys are advantageous because existing master samples can be used to find the desired subjects [81,82], and they are suggested as alternatives to traditional survey methods [83,84]. However, when conducting online surveys, control of unfaithful respondents is important. Therefore, we increased the reliability through an Internet Protocol (IP) check, continuous identical response filtering, and a response-time check by the system. A final total of 400 effective samples were used in the analysis.

Our research staff analyzed the data using tools in SPSS Statistics 23 and Amos 27 (IBM Corp., Armonk, NY, USA). Frequency analysis was conducted to identify the characteristics of the sample, and confirmative factor analysis and discriminant validity analysis were performed to verify the reliability and validity of the variables. Finally, hypotheses one through four were verified through structural equation modeling analysis. On the other hand, although the hypothesis was not established, the difference in the decision-making process in joining screen golf and screen baseball activities within the framework of TAM was verified. In order to analyze the difference between groups, a multiple group analysis using a structural equation model was conducted.

## 4. Findings

### 4.1. Demographic Characteristics of the Respondents

The research team collected a total of 400 valid questionnaires, identifying demographic characteristics such as gender, age, marital status, average household monthly income, educational background, and occupation (see Table 1). In the population participating in screen golf and screen baseball, gender was reported as 80% men and 20% women [85], and the effective sample showed that gender of the respondents was 86.25% men and 13.75% women, indicating a rough correspondence between the effective sample and the overall population of users.

The dominant age category was 40–49 (30.5%), followed by 30–39 (25.0%). Most respondents had obtained higher education, such as university (70.8%), graduate school (20.8%), or college (7.8%). Married people were predominant (64.0%) compared to singles (36.0%). Monthly income above USD 4400 (48.8%) was most prevalent, followed by USD 3600–4400 (20.8%). The dominant occupation category was office workers (35.8%), followed by professionals (23.8%).

### 4.2. Confirmatory Factor Analysis

Results of the confirmatory factor analysis indicate that the fitted index of the measurement model is acceptable: χ^2^/df = 2.018 (χ^2^ = 197.804, df = 98), root mean square residual (RMR) = 0.022, root mean square error of approximation (RMSEA) = 0.051, goodness of fit index (GFI) = 0.941, normed fit index (NFI) = 0.938, incremental fit index (IFI) = 0.968, Tucker-Lewis index (TLI) = 0.960, and comparative fit index (CFI) = 0.967 (see Table 2). Additionally, the standard loadings (≥0.5), conceptual reliability (C.R. ≥ 0.7), and average variance extraction index (AVE ≥ 0.5) of each measurement item were also above the standard values, indicating that concentrated validity was obtained (see Table 2).

Discriminant validity analysis was conducted to test whether the attributes are different for each variable, and was performed utilizing the correlation coefficients of the variables [8]. The correlation coefficients between all variables were found to be within 0.85 recommended levels [86]. The squared values of all correlation coefficients were smaller than the average variance extracted (AVE). Therefore, we could judge that discriminative validity was obtained for all variables (see Table 3).

### 4.3. Hypothesis Testing

The parameter estimates and goodness-of-fit of the structural equation model were evaluated to determine whether the hypothesized structural equation model fit the observed data. The structural equation model’s fit indices (χ^2^/df = 2.09, GFI = 0.938, AGFI = 0.915, RMR = 0.025, CFI = 0.964, TLI = 0.957, RMSEA = 0.052, NFI = 0.934) show good values. Therefore, the interrelationships among the four constructs were verified to test the four proposed hypotheses (see Table 4). To verify the four research hypotheses, a structural equation model was used for the entire sample (*n* = 400).

Therefore, hypothesis verification using a structural equation model was performed on the entire sample (*n* = 400), and three of the four hypotheses were supported. As for perceived ease of use, we could determine a positive effect on perceived usefulness, which supported Hypothesis 1. Hypothesis 2 was not supported because the impact relationship between perceived ease of use and attitude was not significant. Perceived usefulness could be interpreted as a positive effect on attitudes, which supported Hypothesis 3. Attitudes were shown to have a positive effect on intention to use, as demonstrated by testing Hypothesis 4.

In sum, perceived ease of use can be explained as having an indirect effect on attitudes and behaviors as a leading variable of perceived usefulness, supporting the views of some prior studies [43,54,87].

### 4.4. Multiple Group Analysis

Multi-group analysis is a technique used to analyze two or more groups to determine whether the path coefficients between variables in the research model are statistically significant. It is mainly used to compare different samples obtained from a population. Previously conducted hypothesis verification results can be useful for analyzing whether relationships among variables differ between virtual reality sports events. Therefore, the entire sample was divided into screen golf (*n* = 200) and screen baseball (*n* = 200) users, and a multi-group analysis was conducted to ascertain the difference in the influence relationship between variables.

Multi-group analysis performs a three-step process sequentially, proceeding through multiple group confirmatory factor analysis, multi-group path analysis, and multi-group structural equation model analysis. The multiple group confirmatory factor analysis was intended to verify that the measurement scales in this study had measurement uniformity in two groups, which was determined by comparing the difference value between non-constrained and constrained models (χ^2^, df) with a chi-square distribution table.

In the context of this study, it was necessary to verify that each group—screen golf users and screen baseball users—understood the concepts of composition in the survey similarly, which is a pre-validation process for multi-group path analysis. The results of the measurement homogeneity analysis show a difference between the non-constrained model and the constrained model of Δχ^2^ = 9.295 and Δdf = 8, which are presented as Δχ^2^ = 15.51 when *p* = 0.5 and df = 8. The difference of χ^2^ between non-constrained and constrained models is not statistically significant because they appear to be smaller than the values presented in the χ^2^ distribution table. Ergo, there is no issue with measurement tools with regard to measurement uniformity (Table 5).

Multiple group path analysis was performed as the next step, and there was a difference between groups in only one path. Screen Golf Group has not been verified for its perceived ease of use and impact relationship between attitudes, but Screen Baseball Group showed a significant impact relationship between perceived ease of use and attitude (see Table 6). These results enable the judgment that practical usefulness applicable to a real golf course, such as improving golf skills and acquiring golf-related information, is an important influencing factor in forming a positive attitude toward screen golf.

Finally, a multi-group structural equation model analysis was conducted to determine whether the differences in impact relationships specified in the path of the study model were statistically significant. The analysis found that none of the differences between groups on the impact relationships demonstrated in the path were statistically significant (Table 7).

## 5. Discussions

For more than a decade, virtual reality sports games supplemented with cutting-edge information technologies such as AR and VR have grown in popularity. The rising number of patent applications connected to virtual reality sports games, which have been prominent in Korea, provided a good projection of screen golf and screen baseball’s industrialization possibilities. However, at the onset of the COVID-19 pandemic, this set of conditions began to drastically shift. In stark contrast to the continuous rise in screen golf, the decline in screen baseball is noticeable. This study focused on this aspect, and determining what factors contribute to the success and failure of screen golf and screen baseball was established as a research question.

In this regard, changes in trends and social environment transition require a scholarly discussion of why individuals continue to play screen golf while the number of screen baseball users has dramatically declined. However, Korean culture is the only place where virtual reality sports activities, such as screen golf and screen baseball, are widely available, while academic research into the decision-making process for leisure purposes is conspicuously absent. As a result, this study focused on the decision-making process for using screen golf and screen baseball by employing a TAM that is frequently used in understanding the process of consumer adoption of new information technologies.

Virtual reality sports are the product of the integration of cutting-edge information technologies and are continually growing, making them suited to the application of technology-accepting models. Based on a structural equation modeling of survey findings, the suggested research model was found to be suitable, and the hypotheses were partially validated.

Detailed discussion of the analysis results reveals the following:

First, perceived simplicity of use has a considerable positive influence on perceived usefulness. Only perceived usefulness had a substantial influence on attitudes, while the association between perceived ease of use and attitudes was not statistically significant. There was also a significant influence link between attitude and intention to use.

These findings contradict a number of research works that found that reported ease of use and perceived usefulness both promote positive attitude formation, resulting in a strong effect connection between attitude and usage [26,43,88,89,90,91,92,93]. Furthermore, in studies that used TAMs to guide decision-making for food delivery applications, perceived ease of use was found to have a bigger influence on usage than perceived usefulness [8].

TAMs can influence more acceptable hardware and software development for virtual reality sports games, which has both academic and practical ramifications. Ease of use is a key element of IT products, implying that they must provide practical advantages [94], such as obtaining knowledge or increasing skills, in order for users to continue using them. We may assume that the emphasis on the effectiveness of virtual reality sports games has been more significant after the COVID-19 outbreak. With most indoor sports activities being prohibited owing to quarantine measures such as social distance, one factor that may entice individuals to play VR sports games is the utility of continuous training and skill improvement rather than transient enjoyment.

Second, the multi-group analysis results reflect prior research [8] that usefulness is key in VR sports. It also gives a crucial hint regarding the research difficulties. Screen golf demonstrates tremendous technological advancements in meeting the demands of golfers who view utility as a key function. However, it may be required to decide if screen baseball is still at a technological level that does not fulfill that usefulness criteria.

In reality, we can readily and broadly assess that screen golf has technologically advanced to the point where consumers believe they are increasing their particular talents. The use of screen golf is rooted in the improvement in physical skill, and outside golf records may be maintained in real time via a golf application and represented in the training process. In other words, a smart environment in which reality and virtual are coupled, such as that provided by mixed reality (MR) sports, is required. As a consequence, screen golf not only mitigates the loss of individual competence caused by the COVID-19 epidemic, but also helps to strengthen the Korean golf business as a whole.

Screen baseball, on the other hand, has limits as a training tool since it can only simulate a tiny portion of the abilities required in real baseball games, such as batting and pitching. Baseball is a team sport played by two teams of 9 or 10 players who rotate between offense and defense. For attack, many skills such as bunting, base running, and steal must be developed, while for defense, numerous abilities such as throwing and catching must be trained in addition to pitchers’ pitching. The screen baseball stadium’s facilities can only serve extremely limited functions as a genuine baseball training facility. Screen baseball, like screen golf, is thought to require technical progress to the point where it is viable to instruct those who appreciate baseball as a pastime, such as clubs. Screen baseball, in other words, may still be considered as a sport with strong one-time, playfulness, and improvisation.

## 6. Conclusions

Our study team employed a widely recognized technology acceptance model (TAM) to explain the link between perceived ease of use, perceived usefulness, personal attitude, and individual intention in screen golf and baseball decision-making. Structural equation modeling (SEM) confirmed five literature-based assumptions, and 400 effective online survey samples were analyzed. This experiment is especially significant since the findings might address the issue of participants’ perceptions in screen baseball vs screen golf, which has received more attention from Korean society than screen baseball.

Screen baseball-related companies may regain their popularity when the COVID-19 pandemic ends. However, our results help us to deduce technical advances that can maximize the demanded usefulness, a necessity for screen baseball to be recognized as a sport, not just a game. In other words, we suggest that continuous participation can be achieved only when usefulness, i.e., practical training effectiveness, is reinforced by screen baseball. Development of easy-to-use and fun technologies is valuable, but a system should be developed to ensure continuous training for those that desire it, such as developing a class curriculum, high-fidelity reproduction of actual baseball games that can be attacked and defended, and correcting swing and pitching posture.

Despite the theoretical and practical implications that this study advances, the results have limitations, which create a need for follow-up studies. First, it is difficult to find a country where both screen golf and screen baseball are popular, but there is a limitation in the survey’s being confined to a population in Korea. Future research, gathering data from various countries, will provide important results to increase the understanding of cultural and country-specific effects on the use of virtual reality sports games. To our regret, more samples were not secured, even though the results conveyed the need to understand the younger generation for the continued growth in both screen golf and the traditional golf industry. In order to generalize the research results, the survey target should be progressively expanded. Lastly, the types and scopes of virtual reality sports models are becoming excessively diverse. Through this research, we will grow confident in expanding the subject of discussion to include other events, such as running, rowing, table tennis, and cycling, and the generalization of research results across sports, with high growth potential in the future. These should be continuously verified through this type of classification and verification.

## Figures and Tables

**Table 1:** Characteristics of the respondents.

Item		N (%)	Item		N (%)
Gender	Male	345 (86.3)	Monthly Income (US $)	Less than 900	7 (7.8)
	Female	55 (13.8)		900–1800	12 (3.0)
Age	Under 30 years	86 (21.5)		1800–2700	41 (10.3)
	30 s	100 (25.0)		2700–3600	62 (15.5)
	40 s	122 (30.5)		3600–4400	83 (20.8)
	50 s	46 (11.5)		Above 4400	195 (48.8)
	60 s	46 (11.5)	Occupation	Professionals	95 (23.8)
Marriage	Married	256 (64.0)		Office workers	143 (35.8)
	Single	144 (36.0)		Service providers	43 (10.8)
Education	Below high school graduation	3 (0.8)		Self-employed	3 (0.8)
	College university undergraduate	31 (7.8)		Technician	35 (8.8)
	College university graduate	283 (70.8)		Student	42 (10.5)
				Housewives	13 (3.3)
	Post-graduate	83 (20.8)		Others	26 (6.5)

**Table 2:** Results of confirmatory factor analysis.

Constructs		Factor Loading	Variance	AVE	CR
Perceived usefulness	Screen golf (or baseball) helps improve golf skills	0.708 ***	0.257	0.623	0.892
	Screen golf (or baseball) helps you acquire golf-related information	0.726 ***	0.287		
	Screen golf (or baseball) helps reduce the amount of time I spend improving my golf skills	0.723 ***	0.320		
	Screen golf (or baseball) helps reduce the acquisition of golf-related information	0.701 ***	0.325		
	Screen golf (or baseball) saves time to improve my golf skills	0.667 ***	0.316		
Perceived ease of use	Screen golf (or baseball) equipment is easy to use	0.713 ***	0.253	0.678	0.863
	The method of using screen golf- (or baseball)-related equipment is simple and clear	0.775 ***	0.250		
	Screen golf (or baseball) is easy enough for anyone to enjoy	0.722 ***	0.273		
Attitude	I like screen golf (or baseball)	0.793 ***	0.191	0.660	0.906
	I am happy to enjoy screen golf (or baseball)	0.742 ***	0.253		
	I think positively about screen golf (or baseball)	0.665 ***	0.303		
	Screen golf (or baseball) is a valuable leisure activity for me	0.691 ***	0.298		
	Screen golf (or baseball) will bring me goof results	0.693 ***	0.283		
Intention	I will visit the screen golf (or baseball) center as soon as possible	0.790 ***	0.271	0.696	0.873
	I have a plan to visit a screen golf (or baseball)	0.841 ***	0.252		
	I’m sure I’ll be visiting a screen golf (or baseball) center soon	0.807 ***	0.342		

Note: *** *p* < 0.001, AVE = Average Variance Extracted, CR = Construct Reliability.

**Table 3:** Summary of discriminant analysis.

	Perceived Usefulness	Perceived Ease of Use	Attitude	Intention	AVE
Perceived usefulness	**1**				0.623
Perceived ease of use	**0.820** (0.672)	**1**			0.678
Attitude	**0.799** (0.638)	**0.721** (0.520)	**1**		0.660
Intention	**0.706** (0.498)	**0.589** (0.347)	**0.737** (0.543)	**1**	0.696

Note: Bold numbers are correlation coefficients and () are the squares of the correlation coefficients.

**Table 4:** Summary of the tested hypotheses (Screen golf + Screen baseball).

Hypothesized Path		Standardized Coefficient	Standard Error	t	Results
H1	Perceived ease of use → Perceived usefulness	0.832	0.073	11.205 ***	supported
H2	Perceived ease of use → Attitude	0.157	0.115		Not supported
H3	Perceived usefulness → Attitude	0.693	0.125	6.086 ***	supported
H4	Attitude → Intention	0.764	0.072	12.607 ***	supported
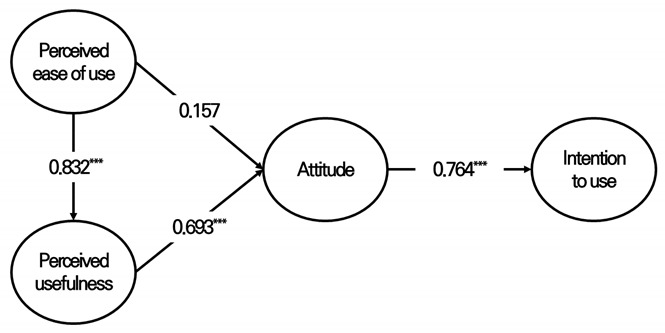					

Note: *** *p* < 0.001.

**Table 5:** Measurement equivalence analysis result.

Model	X^2^	df	GFI	CFI	RMSEA	TLI	Δχ^2^	Sig
Unconstrained *	290.022	196	0.917	0.969	0.035	0.962		
Measurement weights **	299.317	208	0.914	0.970	0.035	0.966	Δχ^2^(8) = 9.295 ***	Not Sig

Note: * Unconstrained model: Models without any constraints. ** Measurement weights model: A model with the same constraints on group factor loadings. *** χ^2^ distribution table (*p* = 0.5), x^2^(8) = 15.51.

**Table 6:** Summary of the tested hypotheses (screen golf vs. screen baseball).

Hypothesized Path		Screen Golf			Screen Baseball		
		Standardized Coefficient	Standard Error	t	Standardized Coefficient	Standard Error	t
H1	Perceived ease of use → Perceived usefulness	0.874	0.122	7.591 ***	0.802	0.091	8.136 ***
H2	Perceived ease of use → Attitude	−0.007	0.259	−0.032	0.268	0.119	2.085 *
H3	Perceived usefulness → Attitude	0.831	0.255	3.937 ***	0.622	0.139	4.502 ***
H4	Attitude → Intention	0.794	0.090	9.975 ***	0.739	0.111	8.142 ***

Note: *** *p* < 0.001, * *p* < 0.05.

**Table 7:** Hypothesis path constraints results.

Hypothesized Path		X^2^	df	Δx^2^	Sig
Unconstrained model		301.715	200		
H1	Perceived ease of use → Perceived usefulness	303.251	201	1.54	not Sig
H2	Perceived ease of use → Attitude	302.536	201	0.82	not Sig
H3	Perceived usefulness → Attitude	303.521	201	1.81	not Sig
H4	Attitude → Intention	301.720	201	0.01	not Sig

## Data Availability

Not applicable.
